# The Patent Flexible Edge

**Published:** 1871-04

**Authors:** W. Geo. Beers


### SELECTED ARTICLES.

### ARTICLE IV.

### "*The Patent Flexible Edge.*"

By W. G. Beers, L.D.S.

Among the numerous so-called "improvements" recently introduced to the dental profession of the United States, and which have been patented, is one called "Straight's Flexible Edge," consisting of a band of flexible rubber placed around the edge of hard vulcanite sets, and for which is claimed, perfection of fit, and infallible adhesion to the palate.

I do not intend attempting to write any elaborate praise or recommendation of the idea, but simply to place the manner of manipulation before the Canadian profession, and letting every one judge for himself by actual experiment of the merits and demerits of the patent. That the plan suggested is not new has been clearly proven, and we would not be surprised to learn that it has been used years ago in Canada just as proposed by the patentee. I remember seeing a very similar combination of hard and flexible rubber in an obturator, at least five years ago, and several now assert that they have even used the rim as patented, for the purpose of securing closer adhesion of hard rubber sets. In the January number of the *Cosmos*, Dr. S. J. McDougall, of Boston, claims to have taught the method several years ago to his students. The question, however, for us, in Canada, is not so much as to priority of discovery, as to the real utility of the rim for practical purposes.

The flexible rubber as sold by the patentee, and which, we believe, can only be obtained from him, is no different to the flexible rubber which can be bought at the depots for the same purpose, except in being divided into strips of the proper width—two sixteenths-of-an-inch. Of course every one can divide the rubber for himself. The process is very simple. First, dissolve some of the ordinary hard rubber in chloform, and keep well corked. After the plaster model is drawn from the impression, trace upon it the exact margin at which the rubber is to terminate at the back of the hard palate, and around the alveolar ridge. It is advisable to make a thorough examination of the condition of the muscles and their attachment to the lips and cheeks, so as to avoid bringing the flexible rim too high. Particular attention must be paid to this, as the rim cannot be trimmed with the same impunity as hard rubber, and in most cases not at all. Immediately where this rim is to lie, the plaster should now be scraped the width and about the depth of the thickness of the rim to be used, so as to ensure a closer fit. The rim being flexible yields, when hard rubber would not, and what would give pain with the latter causes no trouble with the former. The rim is then stuck to the model with the chloform and rubber solution, a little at a time, and very closely and accurately; taking care to close up the minutest hole, lest in packing, any of the hard rubber should get under and destroy the desired elasticity.

Now make the pattern plate of wax or gutta-percha—we prefer the latter—taking care to keep it the sixteenth-of-an-inch from the rim, so as to allow for the usual spread of the hard vulcanite in packing. Fill up the intervening space neatly with wax. The usual process of articulating and grinding on the teeth, may be done at this stage, or before the rim is placed on the model; but any building out of wax or gutta percha, and final finishing preparatory to putting the case in the plaster should be the least part of the proceeding.

The case is then placed in the first part of the flask, *taking particular care to cover the entire rim with plaster except at the inside edge to be united to the hard rubber.* At the back of the case the plaster is brought over and on the rim, and the grooves for the escape of the hard rubber must be cut in the second part of the flask for obvious reasons The rest of the work is the same as in ordinary packing.

It will be observed, that the rim is a considerable obstacle. in finishing up the set, and that great care must be taken not to damage it by the brush wheels in polishing. Felt cones, for pumice stone and soap, as well as whiting or rouge, are preferable to brushes in polishing sets made in this way.

In repairing, or adding the flexible rim to hard rubber, it must be remembered that it cannot be united to hard rubber already vulcanized. Grooves must be cut in the plate, hard rubber added to unite with that already vulcanized, and the flexible rim placed to unite and be vulcanized with the unvulcanized hard vulcanite.

I think that the advantages of accurate fit, &c., obtained by this rim, will be lost in the frequent renewal, which I believe will be found to be necessary, and the possibility of the rim becoming porous and offensive after a few month's wear. We know that obturators made of flexible rubber require frequent renewal; and the general success obtained in fitting vulcanite sets without any flexible rim, will only commend this in cases where all other means to retain artificial sets in the mouth have failed.—*Canada Journal of Dental Science.*

